# A way forward for cancer prevention therapy: personalized risk assessment

**DOI:** 10.18632/oncotarget.27365

**Published:** 2019-12-03

**Authors:** Zhenzhen Zhang, Jeffrey Bien, Motomi Mori, Sonali Jindal, Raymond Bergan

**Affiliations:** ^1^Division of Hematology/Oncology, Knight Cancer Institute, Oregon Health & Science University, Portland, Oregon, USA; ^2^Division of Oncology, Stanford University, Palo Alto, California, USA; ^3^Biostatistics Shared Resource, Knight Cancer Institute, Oregon Health & Science University, Portland, Oregon, USA; ^4^OHSU-PSU School of Public Health, Oregon Health & Science University, Portland, Oregon, USA; ^5^Department of Cell, Developmental & Cancer Biology, Oregon Health & Science University, Portland, Oregon, USA

**Keywords:** cancer, prevention, prevention therapy, multiple risk factors, screening

## Abstract

Cancer is characterized by genetic and molecular aberrations whose number and complexity increase dramatically as cells progress along the spectrum of carcinogenesis. The pharmacologic application of agents in the context of a lower burden of dysregulated cellular processes constitutes an efficient strategy to enhance therapeutic efficacy, and underlies the rationale for using cancer prevention agents in high-risk populations. A longstanding barrier to implementing this strategy is that the risk in the general population is low for any given cancer, many people would have to be treated in order to benefit a few. Therefore, identifying and treating high-risk individuals will improve the risk: benefit ratio. Currently, risk is defined by considering a relatively low number of factors. A strategy that considers multiple factors has the ability to define a much-higher-risk cohort than the general population. This article will review the rationale for evaluating multiple risk factors so as to identify individuals at highest risk. It will use breast and lung cancer as examples, will describe currently available risk assessment tools, and will discuss ongoing efforts to expand the impact of this approach. The high potential of this strategy to provide a way forward for developing cancer prevention therapy will be highlighted.

## INTRODUCTION

Cancer is a disease that develops through a multistep process of carcinogenesis. In the case of advanced cancer, for example metastatic breast cancer, its presence can easily be detected in a given person through widely available technology. However, at this advanced stage, there exists a high number of aberrant genetic, molecular and functional defects that are present within a given individual’s cancer. This underlies the fact that in this situation it is not currently possible to inhibit eventual disease progression and resultant death, even with the application of multiple therapies. In contrast, early in the process of carcinogenesis, the number of aberrant defects that exist within cells is far fewer, and such at risk cells in fact appear morphologically normal. As such, they constitute early events that increase risk for developing cancer in the future, but exist within individuals who do not yet have any clinical evidence of cancer. From a pharmacologic standpoint, such a situation allows one to apply therapy in the context of a lower burden of targets that need to be modulated in order to achieve a given therapeutic outcome. These factors constitute a core rationale for preventing the development of clinical cancer by treating at risk individuals with cancer prevention drugs. An example is the use of single agent tamoxifen, a selective estrogen receptor modulator (SERM), that is able to decrease the chance of a women of ever developing breast cancer by fifty percent among high risk women [1, 2]. This achievement demonstrates the potential power of cancer prevention therapy.

However, several practical barriers impede the attainment of this potential. They include the need to treat many people for long periods of time in order to benefit only a few. Further, there is low tolerance for toxicity, and with any drug, toxicity increases with length of administration. In the case of breast cancer prevention, only 20% of eligible women opt for SERM therapy because of side effects, real or perceived [3].

A logical strategy to begin addressing these barriers involves identifying individuals at higher risk. This creates a more favorable risk: benefit ratio for therapeutic intervention. Further, it decreases the time it takes to assess agent efficacy. Historically, approaches to defining risk have relied upon very few factors, sometimes only age and gender. However, multiple factors have been associated with a given cancer. A strategy that takes into consideration multiple factors therefore has the potential to define risk-enriched cohorts.

Genetic factors account for a portion of individuals’ risk, while demographic and environmental factors substantially contribute. Simultaneously incorporating a combination of genetic factors, personal factors and environmental factors has the potential to more precisely characterize an individuals’ risk, compared to existing approaches. In addition, and importantly, there is a rapidly evolving set of biosensors that provide readouts reflective of factors that either provide measures of early carcinogenic changes, or drivers thereof, thereby having the potential to further refine risk. They are largely driven by emerging technologies that can be applied at the population level and are financially sustainable. Incorporation of all these factors has the ability to identify those whose risk is far greater than that of the general population. The act of targeting screening, counseling and intervention strategies, inclusive of therapeutic, to individuals at the highest risk has the potential to optimally benefit individual outcomes. Further, the diminished clinical readout timeframe will improve the efficiency of testing interventions, inclusive of therapeutic, thereby advancing the field by increasing efficiency.

This review will discuss cancer risk assessment models that take into account multiple factors, will discuss their design, will focus on personalized risk assessment, will focus on lung and breast cancer as examples and will discuss recent advances and future directions. Breast and lung cancer represent the world’s more common types of newly diagnosed cancer [4], and for each cancer type high risk individuals can be identified based on multiple risk factors, but in practice much of this information tends to be underutilized.

## STATISTICAL CONSIDERATIONS FOR DEVELOPMENT OF POPULATION-LEVEL RISK MODELS

**Figure 1:**
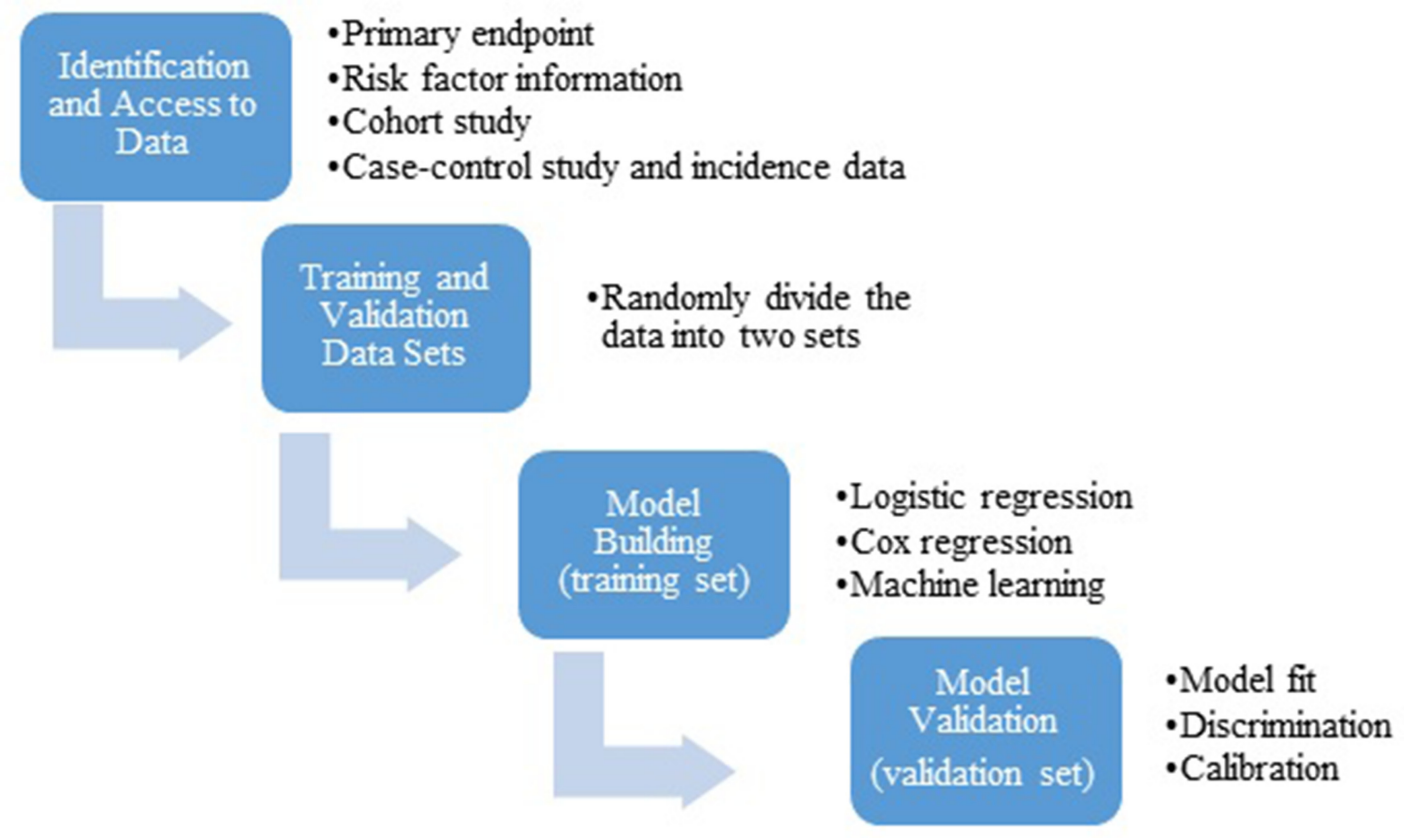
General statistical workflow for development and validation of a risk prediction model.

In order to develop a risk prediction model one must first define what the model is predicting, i.e. its primary endpoint, and the population to which the model will be applied. In most cancer prevention therapy studies, the intended population is the general population, while the primary endpoint is diagnosis of a specific cancer within a certain time period (e.g., diagnosis of breast cancer over the next five years). Appropriate data sets need to be available which contain data on the primary endpoint, known and potential risk factors. Because the cancer incidence is rare in the general population, a data set required for development of a risk prediction model is usually very large (>1 million individuals). In a typical setting, data from a cohort study are used, which contains information on baseline risk factors, outcome (diagnosis of cancer), vital status and follow-up time. In order to evaluate the model performance, data are randomly divided into two sets: a training set and a validation set. The training set is used to develop a model, while the validation set is used to evaluate the model’s performance. In general, the model is developed using a multivariable regression model such as logistic regression for a binary outcome or Cox proportional hazard regression for a censored outcome. The standard variable selection methods (e.g., best subset selection, stepwise procedure) can be used to develop a parsimonious model. In particular, LASSO (least absolute shrinkage and selection operator) is popularly used as a variable selection procedure for high dimensional data such as genome-wide association studies (GWAS) to identify a subset of single nucleotide polymorphisms (SNPs) that are predictive of the cancer risk. The goodness-of-fit of the continuous model was often tested using Hosmer-Lemeshow method. The estimated regression coefficients are then used to generate a predicted risk or probability of the outcome for each subject. More recently machine learning algorithms are used to develop a prediction model, such as random forest and neural network [5], especially for a study with high dimensionality of risk factors and lower than expected events. Regardless of which type and form of the prediction model, the model performance is evaluated using the validation set for discrimination (c-Statistics, ROC AUC), model fit (goodness of fit test) and calibration (expected vs. observed events). Barlow et al. [6] provides an excellent example of the breast cancer risk prediction model following this general strategy using the Breast Cancer Surveillance Consortium (BCSC) (https://www.bcsc-research.org/). The general statistical workflow is illustrated in Figure 1. To be specific, Barlow et al. used data collected from 1,007,600 women participating in the BCSC including seven mammography registries with 2,392,998 eligible screening mammograms (1st step in Figure 1). This is a prospective follow-up study with women at baseline free of breast cancer and newly diagnosed breast cancer cases was ascertained during the follow-up. Barlow et al. separate the data randomly into a training dataset (75% of sample) for risk model construction and a validation dataset (25% of the sample) for validation of the model derived from the training dataset (2nd step in Figure 1). Then, separate logistic regression models were constructed for premenopausal and postmenopausal women in the training dataset using a very stringent level of *P* < 0.0001 for inclusion of covariates, in order to eliminate the concern that small effects can be statistically significant at *P* < 0.05 due to the very large sample size (3rd step in Figure 1). Last, concordance c statistics was calculated for the validation dataset by using the predicted model derived from the training dataset. Goodness-of-fit of the data using Hosmer-Lemeshow test was also used (4 step in Figure 1). After all these steps, the final prediction model was established and validated.

A major limitation for development of a risk prediction model is the availability of data, particularly information related to risk factors. In many cancer types, large cohort study data are unavailable. Instead, case-control study data with the appropriate outcome and risk factor information may be available and can be used to develop a prediction model. The Gail model [7] addressed this challenge by combining case-control study data with cancer incidence data; however, the model still requires appropriate case-control data with relevant risk factor information.

Of highest importance to consider in the context of prevention therapy, one should also note that different risk profiles may give rise to the same probability of cancer incidence, but the biological underpinnings that underlie the different risk factors may in fact be different. Further, this difference in biology may inform a difference in prevention therapy strategy. For example, a 50-year-old woman with a family history of breast cancer and no other risk factor may have a similar probability of a breast cancer as a 50-year-old woman with an extremely dense breast but without a family history of breast cancer. The predicted risk or probability itself may not inform an optimum intervention strategy for each individual; a thorough risk profile and understanding of the potential underlying biological mechanism may be required to develop a personalized prevention therapy strategy.

## STATISTICAL CONSIDERATIONS FOR PREVENTION THERAPY TRIALS

We increasingly need to consider information available from assay methodologies that serve as biosensors, which in turn stem from our rapidly evolving understanding of cancer biology, rather than from evaluation of a population-based database, to be potentially incorporated into a risk prediction model. For initial investigation, a prevention clinical trial study design is a commonly used method. In general, a prevention therapy trial requires a large sample size and longer trial duration because of the very low event rate. For example, the largest prevention trial among women-the Women’s Health Initiative, enrolled 161,808 women and were followed for over 25 years [8]. If there is an accurate risk prediction model, and if we can use it to identify those with the high event rate and those likely to benefit from the prevention therapy, we can design a more efficient trial with a smaller sample size and shorter trial duration. Consider an example of designing a prevention therapy trial using a new biosensor for lung cancer. It is estimated that 10-year risk for lung cancer is 4.0% in the general population and the Bach model’s highest quintile category’s 10-year risk for lung cancer is 10% [9, 10]. Now consider the emerging technology of spectral imaging [11], which uses light-based methods to detect nanoscale macromolecular changes associated with early carcinogenesis. Suppose that those with a cancer associated spectral signature in the oral cavity will have double the risk of developing lung cancer in the next 10 years. Assuming a randomized phase III trial, Table 1 show the sample sizes required to detect 25% reduction in lung cancer risk if the subjects are accrued from the general population (2-year risk of 0.8%), Bach’s highest quintile (2-year risk of 2%), or positive spectral signature (hypothetical 2-year risk of 4%). The benefit of having a strong risk predication model is clear; positive spectral signature, if the risk doubles in that group, will lead to 80% reduction in the sample size.

**Table 1:** Hypothesized sample size for a prevention therapy trial for lung cancer prevention

Risk Group	Power	Per Arm	Total	% Event in Control	% Event in Treatment	Treatment/Control Event Ratio	Alpha	% Reduction in Sample Size
General Population	0.8	27278	54556	0.006	0.008	0.75	0.05	0.0%
Bach Model	0.8	10795	21590	0.015	0.02	0.75	0.05	60.4%
Spectral Imaging	0.8	5301	10602	0.03	0.04	0.75	0.05	80.6%

## REVIEW OF EXISTING CANCER RISK PREDICTION AND ASSESSMENT TOOLS FOR BREAST CANCER

### Background: breast cancer

In 2019, there will be an estimated 268,600 new cases and 41,760 deaths attributed to female breast cancer in the United States [12], making it the most commonly diagnosed cancer and the second most common cause of cancer-related death in women in the country.

### Current risk assessment tools for breast cancer

In the past two decades, several breast cancer risk prediction models have been developed to evaluate a woman’s potential risk for this disease. In addition to the National Cancer Institute (NCI)’s summary on risk assessment models on breast cancer [13], three other systematic reviews have been done on this topic [14–16]. Lee et al. conducted a systematic review and identified 13 risk factors that were consistent throughout models [15]. Van Zitteren et al. conducted a systematic review on genetic risk models [16]. Through applying genetic risk models among a simulated population of 10,000, the study showed the genetic risk models alone based on low susceptibility variants for non-familial breast cancer has the potential to be comparable to that of current breast cancer risk models using clinical and historical risk factor data. More recently, Al-Ajmi et al. reviewed 14 non-clinical/non genetic models and concluded that breast cancer risk models with modifiable risk factors have been well calibrated (i.e. how predicted probabilities agree with observed proportions) but have less ability to discriminate (i.e. the ability to separate individuals into different classes) [14].

#### Pike model and its extended model (Rosner and Colditz model)

The Pike model was developed in 1983 using life-table analysis to estimate the breast cancer risk among breast cancer patients’ mothers and sisters [17]. This model estimates the risk within each decade between 20–70 years of age, depending on the index patient’s age at diagnosis and the laterality of breast cancer. According to the model, first-degree female relatives of premenopausal patients with bilateral disease had higher risk than patients with unilateral disease independent of age at diagnosis. Sisters are at higher risk than mothers. In 1994, a modification of the Pike Model was suggested by Rosner et al. among women participating in the Nurses’ Health Study [18]. The model includes the following variables: age, age at all births, menopause age and menarche age. Later in 1996, Rosner et al., refined the extended Pike model and developed a log-incidence mathematical model, which provided an efficient framework for modeling the effect of lifestyle risk factors on breast cancer incidence, this model also excluded current age from the variable list and a C-statistic = 0.57 was derived [19]. In 2000, Colditz et al. further modified the model by including the following variables: benign breast disease, use of post-menopausal hormones, type of menopause, weight, height and alcohol and the model performance was further improved by showing a C-statistic = 0.64 [20]. Since studies have shown that blood estradiol level in postmenopausal women predict later breast cancer risk, Rosner et al. evaluated the prediction tool adding estradiol levels to the previous log-incidence model [21]. This addition of a serological biomarker significantly improved risk prediction. Rice et al. also updated the Rosner-Coltiz model by including more recently identified historical and behavioral risk factors including adolescent somatotype (body shape and physique type), vegetable intake, breastfeeding, physical activity and predicted percent mammographic density [22] to predict breast cancer risk overall and molecular subtype. This study showed adolescent somatotype and predicted percent mammographic density improved the overall model; among the aggressive subtype of ER-disease, breastfeeding and vegetable intake was associated with improved risk prediction.

#### Gail model and its extended model

The Gail model was developed in 1989 by Dr. Mitchell Gail [7] by combining the risk estimates obtained from a case-control study with age-specific incidence rates (baseline hazards) from the NCI Surveillance, Epidemiology, and End Results (SEER) program. This model is part of the NCI Breast Cancer Assessment Tool (BCRAT) (Table 2), has been validated [23–25], and extended to different race/ethnic groups [26–28].

**Table 2:** Online breast cancer risk assessment model

Model or Tool Name	Website	Risk Factors in the Assessment	Application Population	Comments
The Breast Cancer Risk Assessment Tool (BCRAT) Gail Model	https://bcrisktool.cancer.gov/calculator.html	History of Breast cancer or DCIS or LCIS or previous radiation therapy to the chest or treatment to Hodgkin lymphoma BRCA1 or BRCA2 gene mutation or a genetic syndrome diagnosis Age Race/ethnicity Ever had a breast biopsy number of breast biopsy atypical hyperplasia Age at menarche Age at first live birth of a child Number of first-degree relatives that have had breast cancer	Women 35–74 years old Not applied for women carrying mutation in BRCA1/2, women with a previous history of invasive or *in situ* breast cancer or certain other subgroups	The tool estimates a women’s risk of developing invasive breast cancer over the next 5 years and until age 90 years old. Validated for White, Black, Hispanic and Asian/Pacific Islander women in the U.S.
Breast cancer Surveillance Consortium (BCSC) Risk Calculator	http://tools.bcsc-scc.org/BC5yearRisk/	Age Race/ethnicity Family history of breast cancer in a first-degree female relative History of a breast biopsy with benign breast disease diagnoses if known BI-RADS^®^ breast density assessed by a radiologist	Women 35–74 years old undergoing screening. Not applied to women with previous diagnosis of breast cancer, or DCIS or those who had breast augmentation or mastectomy	In 2015, the BCSC risk calculator has been updated to include benign breast disease diagnoses and to estimate both five-year and ten-year breast cancer risk. Developed and validated in 1.1 million U.S. women and externally validated in the Mayo Mammography Health Study
Tyrer-Cuzick model (IBIS tool) V8	http://www.ems-trials.org/riskevaluator/	Age at menarche Parity Age at first childbirth (if parous) Age at menopause (if postmenopausal) Atypical hyperplasia Locular carcinoma in site Height BMI Family history of breast or ovarian cancer Ashkenazi descent Prior breast biopsy BRCA genes mutations Mammographic density	General population until age 85	This model was first developed from U.K. population. It was validated in Sweden population as well as U.S. population. It can be used for general risk assessment as well as risk of mutation carriers. Mammographic density was recently added into the latest version of the model (V8).
Susan Komen Foundation Risk Factors Table	https://ww5.komen.org/Breastcancer/Breastcancerriskfactorstable.html	Established and probable factors Increases breast cancer risk Decreases breast cancer risk Not related to breast cancer risk (neither increases nor decreases risk) Possible Factors Factors with inconsistent results or insufficient evidence	For general population’s knowledge	The tables lists both factors linked to breast cancer and factors still under study.
CARE model SAS Macro	https://dceg.cancer.gov/tools/risk-assessment/care	Number of breast biopsies Age at menarche in years (non-negative integer years) Number of first degree relatives with breast cancer (non-negative integer counts) Biopsy displays atypical hyperplasia Race Current age Projecting age in years in the set	African American	The model is being updated periodically as new data or research becomes available.
Breast and Ovarian Analysis of Disease Incidence and Carrier Estimation Algorithm (BOADICEA)	https://pluto.srl.cam.ac.uk/cgi-bin/bd3/v3/userReg2.web2.cgi	Pedigree number Clinical history (sex and status, age or age at death, year of birth), age at breast cancer diagnosis, age at ovarian cancer diagnosis, age at pancreatic cancer diagnosis, genetic testing Breast cancer pathology (ER, PR, HER2, CK14, CK5/6)	General population and individuals with family history	BOADICEA can be used to predict BRCA1/2 mutation carrier probabilities and breast cancer as well as ovarian cancer risks at specific future ages

The earliest Gail Model took into account of age at menarche, age at the time of first live birth, the number of first degree relatives with breast cancer, the number of breast biopsies; later, history of breast cancer, ductal carcinoma *in situ* (DCIS) or lobular carcinoma *in situ* (LCIS), BRCA1/2 mutation status, current age, biopsy displaying atypical hyperplasia and race were added into the model.

The Gail model was also developed based on estrogen receptor (ER) status in postmenopausal women. Chlebowski et al. utilized data from the WHI cohort and compared the model’s performance for ER-positive and ER-negative breast cancer and showed that the Gail model could identify populations at increased risk for ER+ but not ER- cancer [29].

Other extensions of Gail model include modification made by Clause et al. to consider women with first degree family history of ovarian cancer [30], and by Jonker et al. [31] to estimate the familial cluster of breast cancer to include BRCA1, BRCA2 and a hypothetical BRCAu either as dominant or recessive variant. Jonker’s model is comparable to Claus et al.’s and the dominant and the recessive model provided similar estimates.

The performance of the Gail breast cancer risk prediction model among the non-white population remains understudied in the US, though there have been some attempts to tailor it to specific non-white population [27]. One modified Gail model, termed as AABCS (Asian American breast cancer study) estimated absolute risk separately for Chinese, Japanese, Filipino, Hawaiian, Pacific Islander, and Asian women [27]. The AABCS model was calibrated to ethnicity-specific incidence rates and in these groups demonstrated better performance than the Gail (BCRAT) model for counseling Asian Pacific American women. The Gail model was also calibrated for Hispanic women, the Hispanic risk model (HRM). The HRM model was found to overestimate the risk for foreign-born women compared to US-born women and suggested further evaluation of its validity [28]. The CARE model targeted the African American population, was modified from the Gail model [26], and used data collected from African American case and control subjects in the Women’s Contraceptive and Reproductive Experiences (CARE) Study. Recently, Boggs et al. developed a new model for African American women based on 10 years of follow-up from the Black Women’s Health Study (BWHS) [32]. The new BWHS model included family history, previous biopsy, BMI at age 18 years, age at menarche, age at first birth, oral contraceptive use, bilateral oophorectomy, estrogen plus progestin use and height. This new model was well calibrated with higher discriminatory accuracy among younger African American women less than age 50 years.

Finally, it is well recognized that breast cancer patterns vary between countries. Multiple extension models of the Gail model have therefore been tailored to different counties, including Japan [33], Korea (KoBCRAT model) [34, 35], Thailand [36], Italy (IT-GM model) [37, 38], the Czech Republic [39], Sweden [40], Sudan [41] and Nigeria [42]. All such extensions have been shown to improve performance.

Dense breast tissue is known to be a strong risk factor for breast cancer development [43]. Chen et al. incorporated breast density from mammogram results as a variable into the Gail model. This adjusted model predicted higher risks when compared against the Gail model among women with higher breast density; however, the model provided average risk prediction in various age groups similar to the Gail model, suggesting that the new model has modest improvement in discriminatory power [44]. Another study by Tice et al. using the BCSC data, however, showed breast density incorporated into a breast cancer prediction model can improve the estimated 5-year risk of invasive breast cancer [45].

#### Tyrer-Cuzick model

In 2004, out of the International Breast cancer Intervention Study (https://www.ibis-trials.org/), Tyrer and Cuzick et al. developed a breast cancer risk prediction model (Tyrer-Cuzick model or IBIS tool, Table 2) incorporating the BRCA genes and a panel of more comprehensive personal risk factors [46]. The model includes classic breast cancer risk factors as well as genetic testing results. It has been validated in many studies [47–49] and the most current version incorporated breast mammographic density.

#### Breast Cancer Surveillance Consortium (BCSC) risk prediction model

Scientists participating in the BCSC (Barlow et al.) developed the risk calculator to estimate both 5-year and 10-year breast cancer risk [6]. For premenopausal breast cancer, age, breast density, family history of breast cancer and a prior breast procedure were significant risk factors; for postmenopausal breast cancer, age, breast density, race, ethnicity, family history of breast cancer, a prior breast procedure, BMI, natural menopause, hormone therapy and a prior false-positive mammogram were risk factors. It was validated among 1.1 million women undergoing mammography across the U.S., with 18,000 diagnosed with invasive breast cancer. The BCSC Risk Calculator (Table 2) has been externally validated in the Mayo Mammography Health Study [50].

#### Inclusion of Single Nucleotide Polymorphisms (SNPs) into risk prediction and hypergeometric polygenic model

With the development of GWAS, there has been increasing interest in adding SNPs identified from GWAS to breast cancer risk prediction models. Supplementary Table 1 summarizes the risk prediction models for breast cancer incorporating SNPs in the models.

Wachold et al. evaluated 10 common genetic variants that, when added to traditional risk factors, were used to predict breast cancer risk [51]. The results showed that adding additional genetic variant information only modestly improved the risk prediction models’ performance. Further stratifying breast cancer into its molecular subtypes, Husing et al. evaluated the predictive capacity of GWAS-identified SNPs alone and in combination with traditional risk factors among breast cancer cases with different hormone receptor status [52]. Through AUC analysis, increasing the numbers of genetic variants does steadily increase the discriminatory ability in breast cancer risk prediction; however, the overall effect size is small. Importantly, the discrimination performs better in receptor-positive cases.

Dite et al. conducted age group stratified analyses among cases and controls aged 35–49 years in the Australian Breast Cancer Family Registry to evaluate whether young age groups would have better performance than older age group [53]. Through calculating the AUC, the authors found among women aged 35–39 years, the AUC improved from 0.61 to 0.65 after including seven SNPs; among women aged 40–49 years, the AUC improved from 0.61 to 0.63. The overall AUC improved from 0.58 to 0.61. The data showed incorporating SNPs into the BRCAT model improved the model performance especially among women aged 35–49 years.

Jupe et al. developed a polyfactorial risk model (PFRM) to predict sporadic breast cancer risk among a Caucasian population [54]. This model included 5 clinical risk factors and 22 SNPs including 19 genes with 6 genes specifically involved in the regulation of steroid metabolism. This model was found to outperform the Gail model almost 2 fold in terms of 5-year and lifetime risk prediction. The model was developed based on 5,022 Caucasian women and validated in another cohort of 1,193 women.

McCarthy et al. evaluated the performance of the BCRAT and the combined BCRAT+SNPs model using a cohort of African American and white women [55]. Agreement between the BCRAT and the BCRAT+SNPs model was low for identifying high-risk women. Adding SNPs had the greatest prediction impact among African Americans, with 33% identified as high-5-year risk by the combined model compared against 12.4% identified by the BCRAT. Among African Americans, 21% were reclassified as having high-5-year risk, while 10% of white women were reclassified. This study showed evidence that SNPs are especially valuable among African Americans for use in breast cancer risk prediction. Likewise, combining together SNPs with clinical risk factors improves risk prediction in both Chinese and Japanese populations [56, 57].

Van Veen et al. evaluated a panel of 18 SNPs (SNP18) in combination with classic risk assessed by the Tyrer-Cuzick model [58]. This study found adding SNP18 provided a better risk stratification among women in both the lower and higher risk groups [58].

Antoniou et al. developed a hypergeometric polygenic BOADICEA model for populations with a known family history of breast or ovarian cancer by incorporating BRCA1 and BRCA2 mutations, as well as the joint multiplicative effects of a polygenic component of multiple genes of small effect [59]. Later, BOADICEA model was updated using data from two UK breast cancer studies and family data from BRCA1/2 carriers [60]. The BOADICEA is applied to individuals with any family history to predict their gene mutation probabilities and cancer risk. Later, Lee et al. further updated the BOADICEA model extending its capabilities, making it easier to use in clinics with more accurate predictions, and updated the web interface to be available for general use [61].

In addition to the use of breast cancer or BRCA1/2 mutation status as the primary variable, Crooke et al. developed a model for looking at the kinetic effect of genetic variants of the enzymes CYP1A1, CYP1B1, and COMT as well as phenotype factors on the production of the main carcinogenic estrogen metabolite 4-hydroxyestradiol (4-OHE(2)), expressed as an AUC metric (4-OHE(2)-AUC) [62]. This model showed women at higher 4-OHE(2)-AUC level are at greater risk developing breast cancer among both pre- and post-menopausal women.

To evaluate whether polygenic risk score (PRS) or breast density from BI-RADS is independent risk factor for breast cancer or not, Vachon et al. evaluated PRS and breast density into the BCSC risk prediction model and found that PRS added independent information (*P* < .001) to the BCSC model and improved discriminatory accuracy [50]. Shieh et al. evaluated the BCSC risk model in combination with a PRS compared of 83 SNPs identified from GWAS studies [63]. Using data from a nested case-control study within a screening cohort, the study found the PRS, family history and breast density remained strong risk factors, a combined model including the BCSC risk factors with PRS improved the BCSC model to AUROC 0.65 from 0.62. The BCSC-PRS model classified 18% of cases as high-risk compared with 7% by BCSC model. This study further suggested BCSC-PRS model’s better prediction ability.

### Summary of breast cancer risk prediction models

Breast cancer prediction models are developed and refined over years, tailored to both the general population and specific populations, with dynamic adjustments given our evolving understanding of pathophysiology, risk factors, and detection technology development. The calibration performance and discrimination performance vary across models. There is no universal model to predict breast cancer risk, though among different populations, a constellation of different models exist and are undergoing constant refinement. Key variables that are used to predict breast cancer occurrence include: multiple ages (current age, age at menarche, age at 1st child birth), race/ethnicity, family history of breast cancer among first degree female relatives, history of breast biopsy, breast density, genetic factors including BRCA gene mutations and the factors may be expanded/refined with more research update. Efforts that leverage orthogonally informative sources of information, inclusive of history, high dimensional genetic analysis, and an emerging panel of biosensors, while striving for simplicity of models will be at the forefront of effectiveness.

## REVIEW OF EXISTING CANCER RISK PREDICTION AND ASSESSMENT TOOL FOR LUNG CANCER

### Background: lung cancer

Lung cancer is the leading cause of cancer-related mortality both in the U.S. and worldwide, for both men and women. In 2018 in the U.S. there were over 234,000 new cases of lung cancer, accounting for 25.3% of all cancer deaths [64]. Small cell lung cancer (SCLC) and non-small cell lung cancer (NSCLC) account for the vast majority of lung cancer diagnoses, comprising approximately 15% and 85%, respectively [65]. If detected at an early stage, lung cancer can be cured through local modality-based therapy.

The single most important risk factor associated with the development of lung cancer is tobacco smoking [66]. Tobacco use patterns, in response to control efforts are largely responsible for the decreased incidence in the U.S. However, the success of such control efforts are varied worldwide. In addition to tobacco smoke exposure, other factors associated with the development of lung cancer include air pollution, radiation, coal smoke, indoor fuel burning, other organic compounds, industrial exposure to asbestos or mining of metals, and exposure to radon [65].

### Lung cancer screening

The natural history of lung cancer is such that there is often a long asymptomatic preclinical phase. This scenario is not well suited for using clinical signs for early detection, but does provide the requisite opportunity for early detection in the preclinical phase. Symptoms such as cough, hemoptysis, dyspnea and chest pain often reflect invasion into local or distant structures, often signaling advanced disease [67]. However, the epidemiology of lung cancer makes it a particularly attractive candidate for screening given its significant prevalence, identifiable risk factors and the ability to cure early stage disease.

Although several modalities for lung cancer screening have been considered, including chest radiography and sputum cytology testing, the only modality that has been prospectively shown to reduce mortality in a randomized trial is low-dose computed tomography (LDCT). This benefit was proven in the National Lung Screening Trial (NLST), which demonstrated a relative reduction in mortality of 20% when using annual screening by LDCT compared to chest radiographs [68]. The study evaluated individuals between 55–74 years old with at least 30 pack-years smoking history, and for non-current smokers, cessation within 15 years. Based largely on this study, LDCT screening for relevant cohorts is recommended by the American Cancer Society, National Comprehensive Cancer Network, American Society of Clinical Oncology, the US Preventative Services Task Force and other groups. However, some groups, such as the American Academy of Family Physicians, conclude that there is insufficient evidence to recommend for or against screening. If all currently eligible individuals in the U.S, were screened according to these criteria, a 2018 estimate concluded that 12,000 lives per year could be saved [69].

### Poor uptake of screening

Despite the compelling effectiveness data and guidelines advocating for broader uptake of LDCT for screening eligible populations, a 2017 study found that only 3–4% of eligible individuals are undergoing the recommended screening [70]. There are several underlying reasons. Firstly, lung cancer screening itself does not come without risks. While LDCT demonstrated a reduction in mortality, the screening process exposes all patients to radiation, and there was a high incidence of false positives among positive screening results. Further, half of lung cancer cases continue to occur outside of the screening-eligible population [71]. Positive screening findings caused significant anxiety and stress, and many exposed patients to invasive and potentially unnecessary procedures such as biopsy, bronchoscopy, or surgery. Additionally, over diagnosis itself is a risk, a situation in which the presence of a cancer is detected early, but the natural history of the cancer detected may not have gone on to cause symptoms or limit life expectancy. Further, patients identified as high-risk for development of lung cancer have concordant increased risk for related comorbid conditions, such as chronic obstructive pulmonary disease, coronary artery disease or cerebrovascular disease among many others [72]. These comorbid conditions not only compete with the potential development of lung cancer as conditions that can adversely impact health, they add significantly to the risk of conducting invasive procedures in pursuit of positive initial LDCT findings. Therefore, the patients identified as the most likely to benefit from early detection and early intervention may also be the poorest candidates for said early intervention based on their comorbidity profiles. Finally, there are large financial implications associated with screening and the costs of following up on the high rate of false positive findings.

Taken in concert, the demonstrable benefits in mortality achievable with lung cancer screening, the high false-positive rate of said screening, and relatively low uptake of such screening despite guideline recommendations all suggest that development of tools to optimize screening patient populations by improving specificity could result in improved uptake of screening and therefore lives saved.

### Current risk assessment tools for lung cancer

Current screening recommendations based on the NLST data identify candidates for screening based solely on their age and past cigarette exposure in the form of pack-years. One means of limiting over diagnosis would be to apply increasingly precise, targeted-screening protocols towards populations with an enriched disease burden. As a parallel to breast cancer, the development of risk-assessment tools may assist with identifying individuals with the highest pre-test probability of disease. In fact, an analysis of the NLST data that divides the screened individuals into quintiles based on perceived 6-year risk of death from lung-cancer demonstrates that those in the highest quintile of risk contained nearly 90% of the screen-prevented lung cancer deaths [73]. Within the highest quintile, the number needed to screen to prevent 1 lung cancer death was 161.

Several subsequent risk-assessment tools have been developed or are under development that incorporate additional historical and demographic information beyond smoking history and age, such as race, ethnicity, education, obesity, history of cancer, and other comorbidities, or even other proposed screening tests or biomarkers which could improve the operating characteristics of the LDCT test to more specifically predict risk of lung cancer development. Despite the number of risk assessment tools, they are difficult to validate in real-world patient populations. A recent analysis of nine accepted models compared their relative performance characteristics by retrospectively using the NLST and the Prostate, Lung, Colorectal, and Ovarian Cancer Screening Trial (PLCO) data [74]. By assessing these models based on their calibration, discrimination, and clinical usefulness, the authors found that the PLCOm2012, Bach, and Two-Stage Clonal Expansion incidence models had the best characteristics, outperforming the original NLST entry criteria in both sensitivity and specificity [9, 75, 76]. However, this concept has not yet been proven in a prospective randomized trial in the same way the original NLST demonstrated a mortality benefit.

The concept of tying a separate test modality, such as a biomarker, to complement imaging (specifically LDCT) has been a tantalizing prospect as a means to orthogonally modify an individual’s candidacy for screening or to interpret indeterminate screening results. However, there are numerous biomarker candidates that are in a variety of stages of development, and none have been prospectively evaluated in a randomized clinical context to assess their real-world performance characteristics, let alone integrated with LDCT to see if they improve upon the ability to detect early cases of lung cancer and prevent mortality. Acknowledging this limitation, a 2016 simulation performed by Kong, et. al examines the prospect of a combination theorized biomarker and LDCT strategy for screening in the context of cost-effectiveness [77]. However, because the biomarkers assessed in this study are theoretical, the generalizability of the conclusions are limited.

To further investigate the premise of better identifying candidates for screening with biomarkers, the 2018 INTEGRAL study evaluated the performance of a panel of biomarkers [cancer antigen 125 (CA-125), carcinoembryonic antigen (CEA), cytokeratin-19 fragment (CYFRA 21–1), and precursor surfactant B (Pro-SFTPB] with LDCT [78, 79]. This panel was developed based on a cohort of patients at high risk of lung cancer, and then blindly validated in a separate cohort of patients of 63 patients with lung cancer and 90 matched controls. The results do demonstrate improvement in the AUC of patients who develop lung cancer when using the biomarker panel compared to the smoking status alone as per the original NLST population. SNPs have been also incorporated into lung cancer prediction models and Supplementary Table 2 summarized the risk prediction models for lung cancer with SNPs included as one of the predictors.

The potential application of a robust multifactorial risk assessment tool for lung cancer risk could be well suited to identify ideal candidates for lung cancer screening programs. Taken a step further, a robust tool would similarly identify strong candidates for prevention therapy strategy, in a similar vein to the above proposed strategies with regards to breast cancer.

New biosensor technology is increasingly able to identify high-risk populations, narrowing down prevention trial sample size. For example, special imaging is a fast developing field for early diagnosis. The basic principle of spectral analysis is that the epithelial surface exposed to the cohesive laser will reflect a unique spectral pattern. The nature of the pattern is determined by the intracellular nanoscale macromolecular structure, especially chromatin. Chromatin structure is one of the earliest and most common changes that can drive cancer. In upper respiratory tracts, including lung and several organ types, all of these organs are at risk of “field carcinogenic” effects [80]. Assess the risk of a nearby organ area, such as the mouth, can predict the risk of lung cancer. Consider smokers and lung cancer, people who smoke expose cells in their oral cavity to smoke at the same time they expose their lungs to it. Changes in the spectral signature of oral cells are seen coincident with changes in lung epithelial cells. As a non-invasive method, oral cell DNA is readily available in contrast to DNAs from the lung. Some of the oral DNA adducts have been identified in lung DNA from smokers and changes in DNA can be routinely quantified by spectral imaging technique [81]. We expect the intensity of the spectral characteristics of oral epithelial cell DNA increases with the increase in the risk of lung cancer, therefore, it can be readily used into lung cancer risk prediction model to improve identifying the high-risk cancer population.

## CONCLUSIONS

Cancer is not predicted by a single factor. A combination of non-clinical, clinical, and genetic risk factors together provide a more comprehensive and accurate assessment of risk for each individual. Efficient prevention therapy strategies will need to rely on such comprehensive risk assessment tools for targeted intervention and for effective cancer prevention strategies that are both sustainable and acceptable. The landscape of biomarker technology is extremely dynamic, with many promising new candidates, covering a spectrum of operating characteristics. Therefore, the development of risk prediction models for prevention therapy will need to evolve in a rational manner in order to incorporate the many factors that contribute to risk. Future prevention therapy trials will increasingly rely on the continued development of increasingly robust risk assessment tools to not only quantify risk, but to also determine the biological basis of that risk, and thus the type of intervention strategy.

## SUPPLEMENTARY MATERIALS







## Abbreviations

AABCS: Asian American Breast Cancer Study
AUC: area under the ROC curve
BCRAT: Breast Cancer Assessment Tool
BCSC: Breast Cancer Surveillance Consortium
BMI: Body Mass Index
BOADICEA: Breast and Ovarian Analysis of Disease Incidence and Carrier Estimation Algorithm
BWHS: Black Women’s Health Study
CARE: Contraceptive and Reproductive Experiences
DCIS: ductal carcinoma *in situ*
ER: estradiol receptor
HER2: human epidermal growth factor receptor 2
GWAS: genome-wide association studies
LASSO: least absolute shrinkage and selection operator
LCIS: lobular carcinoma *in situ*
LDCT: low-dose computed tomography
NCI: National Cancer Institute
NLST: National Lung Screening Trial
NSCLC: non-small cell lung cancer
PFRM: polyfactorial risk model
PLCO: Prostate, Lung, Colorectal, and Ovarian Cancer Screening Trial
ROC: receiver operating characteristic
SCLC: small cell lung cancer
SEER: Surveillance, Epidemiology, and End Results
SERM: selective estrogen receptor modulator
SNPs: single nucleotide polymorphisms
WHI: Women’s Health Initiative
