# Supplementary material for: A way forward for cancer prevention therapy: personalized risk assessment
Source: Oncotarget. 2019 Dec 3;10(64):6898–912. doi: 10.18632/oncotarget.27365 (PMC6901339; doi:10.18632/oncotarget.27365)
Supplement: Supplementary file 1 [file oncotarget-10-6898-s001.pdf]

A way forward for cancer prevention therapy: personalized risk assessment

SUPPLEMENTARY MATERIALS

Supplementary Table 1: Current risk assessment model incorporating genetic risk factors for breast cancer risk prediction. See Supplementary Table 1

Supplementary Table 2: Current risk assessment model incorporating genetic risk factors for lung cancer risk prediction. See Supplementary Table 2

REFERENCES

1. van Zitteren M, van der Net JB, Kundu S, Freedman AN, van Duijn CM, Janssens AC. Genome-based prediction of breast cancer risk in the general population: a modeling study based on meta-analyses of genetic associations. Cancer Epidemiol Biomarkers Prev. 2011; 20:9–22. https://doi.org/10.1158/1055-9965.EPI-10-0329. [PubMed]
2. Claus EB, Risch N, Thompson WD. The calculation of breast cancer risk for women with a first degree family history of ovarian cancer. Breast Cancer Res Treat. 1993; 28:115–20. https://doi.org/10.1007/bf00666424. [PubMed]
3. Jonker MA, Jacobi CE, Hoogendoorn WE, Nagelkerke NJ, de Bock GH, van Houwelingen JC. Modeling familial clustered breast cancer using published data. Cancer Epidemiol Biomarkers Prev. 2003; 12:1479–85. [PubMed]
4. Darabi H, Czene K, Zhao W, Liu J, Hall P, Humphreys K. Breast cancer risk prediction and individualised screening based on common genetic variation and breast density measurement. Breast Cancer Res. 2012; 14:R25. https://doi.org/10.1186/bcr3110. [PubMed]
5. Tyrer J, Duffy SW, Cuzick J. A breast cancer prediction model incorporating familial and personal risk factors. Stat Med. 2004; 23:1111–30. https://doi.org/10.1002/sim.1668. [PubMed]
6. Vachon CM, Pankratz VS, Scott CG, Haeberle L, Ziv E, Jensen MR, Brandt KR, Whaley DH, Olson JE, Heusinger K, Hack CC, Jud SM, Beckmann MW, et al. The contributions of breast density and common genetic variation to breast cancer risk. J Natl Cancer Inst. 2015; 107:dju397. https://doi.org/10.1093/jnci/dju397. [PubMed]
7. Wacholder S, Hartge P, Prentice R, Garcia-Closas M, Feigelson HS, Diver WR, Thun MJ, Cox DG, Hankinson SE, Kraft P, Rosner B, Berg CD, Brinton LA, et al. Performance of common genetic variants in breast-cancer risk models. N Engl J Med. 2010; 362:986–93. https://doi.org/10.1056/NEJMoa0907727. [PubMed]
8. Husing A, Canzian F, Beckmann L, Garcia-Closas M, Diver WR, Thun MJ, Berg CD, Hoover RN, Ziegler RG, Figueroa JD, Isaacs C, Olsen A, Viallon V, et al. Prediction of breast cancer risk by genetic risk factors, overall and by hormone receptor status. J Med Genet. 2012; 49:601–8. https://doi.org/10.1136/jmedgenet-2011-100716. [PubMed]
9. Dite GS, Mahmoodi M, Bickerstaffe A, Hammet F, Macinnis RJ, Tsimiklis H, Dowty JG, Apicella C, Phillips KA, Giles GG, Southey MC, Hopper JL. Using SNP genotypes to improve the discrimination of a simple breast cancer risk prediction model. Breast Cancer Res Treat. 2013; 139:887–96. https://doi.org/10.1007/s10549-013-2610-2. [PubMed]
10. Jupe ER, Dalessandri KM, Mulvihill JJ, Miike R, Knowlton NS, Pugh TW, Zhao LP, DeFreese DC, Manjeshwar S, Gramling BA, Wiencke JK, Benz CC. A steroid metabolizing gene variant in a polyfactorial model improves risk prediction in a high incidence breast cancer population. BBA Clin. 2014; 2:94–102. https://doi.org/10.1016/j.bbacli.2014.11.001. [PubMed]
11. McCarthy AM, Armstrong K, Handorf E, Boghossian L, Jones M, Chen J, Demeter MB, McGuire E, Conant EF, Domchek SM. Incremental impact of breast cancer SNP panel on risk classification in a screening population of white and African American women. Breast Cancer Res Treat. 2013; 138:889–98. https://doi.org/10.1007/s10549-013-2471-8. [PubMed]
12. Dai J, Hu Z, Jiang Y, Shen H, Dong J, Ma H, Shen H. Breast cancer risk assessment with five independent genetic variants and two risk factors in Chinese women. Breast Cancer Res. 2012; 14:R17. https://doi.org/10.1186/bcr3101. [PubMed]
13. Sueta A, Ito H, Kawase T, Hirose K, Hosono S, Yatabe Y, Tajima K, Tanaka H, Iwata H, Iwase H, Matsuo K. A genetic risk predictor for breast cancer using a combination of low-penetrance polymorphisms in a Japanese population. Breast Cancer Res Treat. 2012; 132:711–21. https://doi.org/10.1007/s10549-011-1904-5. [PubMed]

14. van Veen EM, Brentnall AR, Byers H, Harkness EF, Astley SM, Sampson S, Howell A, Newman WG, Cuzick J, Evans DGR. Use of Single-Nucleotide Polymorphisms and Mammographic Density Plus Classic Risk Factors for Breast Cancer Risk Prediction. JAMA Oncol. 2018; 4:476–82. https://doi.org/10.1001/jamaoncol.2017.4881. [PubMed]
15. Antoniou AC, Pharoah PP, Smith P, Easton DF. The BOADICEA model of genetic susceptibility to breast and ovarian cancer. Br J Cancer. 2004; 91:1580–90. https://doi.org/10.1038/sj.bjc.6602175. [PubMed]
16. Antoniou AC, Cunningham AP, Peto J, Evans DG, Lalloo F, Narod SA, Risch HA, Eyfjord JE, Hopper JL, Southey MC, Olsson H, Johannsson O, Borg A, et al. The BOADICEA model of genetic susceptibility to breast and ovarian cancers: updates and extensions. Br J Cancer. 2008; 98:1457–66. https://doi.org/10.1038/sj.bjc.6604305. [PubMed]
17. Lee AJ, Cunningham AP, Kuchenbaecker KB, Mavaddat N, Easton DF, Antoniou AC, Consortium of Investigators of Modifiers of B, Breast Cancer Association C. BOADICEA breast cancer risk prediction model: updates to cancer incidences, tumour pathology and web interface. Br J Cancer. 2014; 110:535–45. https://doi.org/10.1038/bjc.2013.730. [PubMed]
18. Crooke PS, Justenhoven C, Brauch H, Consortium G, Dawling S, Roodi N, Higginbotham KS, Plummer WD, Schuyler PA, Sanders ME, Page DL, Smith JR, Dupont WD, et al. Estrogen metabolism and exposure in a genotypic-phenotypic model for breast cancer risk prediction. Cancer Epidemiol Biomarkers Prev. 2011; 20:1502–15. https://doi.org/10.1158/1055-9965.EPI-11-0060. [PubMed]
19. Shieh Y, Hu D, Ma L, Huntsman S, Gard CC, Leung JW, Tice JA, Vachon CM, Cummings SR, Kerlikowske K, Ziv E. Breast cancer risk prediction using a clinical risk model and polygenic risk score. Breast Cancer Res Treat. 2016; 159:513–25. https://doi.org/10.1007/s10549-016-3953-2. [PubMed]
20. Claus EB, Risch N, Thompson WD. Autosomal dominant inheritance of early-onset breast cancer. Implications for risk prediction. Cancer. 1994; 73:643–51. https://doi.org/10.1002/1097-0142(19940201)73:3%3C643::aid-cncr2820730323%3E3.0.co;2-5. [PubMed]
21. Antoniou AC, Pharoah PD, McMullan G, Day NE, Stratton MR, Peto J, Ponder BJ, Easton DF. A comprehensive model for familial breast cancer incorporating BRCA1, BRCA2 and other genes. Br J Cancer. 2002; 86:76–83. https://doi.org/10.1038/sj.bjc.6600008. [PubMed]
22. Berry DA, Iversen ES Jr, Gudbjartsson DF, Hiller EH, Garber JE, Peshkin BN, Lerman C, Watson P, Lynch HT, Hilsenbeck SG, Rubinstein WS, Hughes KS, Parmigiani G. BRCAPRO validation, sensitivity of genetic testing of BRCA1/BRCA2, and prevalence of other breast cancer susceptibility genes. J Clin Oncol. 2002; 20:2701–12. https://doi.org/10.1200/JCO.2002.05.121. [PubMed]
23. Garcia-Closas M, Gunsoy NB, Chatterjee N. Combined associations of genetic and environmental risk factors: implications for prevention of breast cancer. J Natl Cancer Inst. 2014; 106:dju305. https://doi.org/10.1093/jnci/dju305. [PubMed]
24. Mavaddat N, Pharoah PD, Michailidou K, Tyrer J, Brook MN, Bolla MK, Wang Q, Dennis J, Dunning AM, Shah M, Luben R, Brown J, Bojesen SE, et al. Prediction of breast cancer risk based on profiling with common genetic variants. J Natl Cancer Inst. 2015; 107:djv036. https://doi.org/10.1093/jnci/djv036. [PubMed]
25. Maas P, Barrdahl M, Joshi AD, Auer PL, Gaudet MM, Milne RL, Schumacher FR, Anderson WF, Check D, Chattopadhyay S, Baglietto L, Berg CD, Chanock SJ, et al. Breast Cancer Risk From Modifiable and Nonmodifiable Risk Factors Among White Women in the United States. JAMA Oncol. 2016; 2:1295–302. https://doi.org/10.1001/jamaoncol.2016.1025. [PubMed]
26. Chan CHT, Munusamy P, Loke SY, Koh GL, Yang AZY, Law HY, Yoon CS, Wong CY, Yong WS, Wong NS, Ng RCH, Ong KW, Madhukumar P, et al. Evaluation of three polygenic risk score models for the prediction of breast cancer risk in Singapore Chinese. Oncotarget. 2018; 9:12796–804. https://doi.org/10.18632/oncotarget.24374. [PubMed]
27. Mavaddat N, Michailidou K, Dennis J, Lush M, Fachal L, Lee A, Tyrer JP, Chen TH, Wang Q, Bolla MK, Yang X, Adank MA, Ahearn T, et al. Polygenic Risk Scores for Prediction of Breast Cancer and Breast Cancer Subtypes. Am J Hum Genet. 2019; 104:21–34. https://doi.org/10.1016/j.ajhg.2018.11.002. [PubMed]
28. Spitz MR, Etzel CJ, Dong Q, Amos CI, Wei Q, Wu X, Hong WK. An expanded risk prediction model for lung cancer. Cancer Prev Res (Phila). 2008; 1:250–4. https://doi.org/10.1158/1940-6207.CAPR-08-0060. [PubMed]
29. Deng L, Kimmel M, Foy M, Spitz M, Wei Q, Gorlova O. Estimation of the effects of smoking and DNA repair capacity on coefficients of a carcinogenesis model for lung cancer. Int J Cancer. 2009; 124:2152–8. https://doi.org/10.1002/ijc.24149. [PubMed]
30. Raji OY, Agbaje OF, Duffy SW, Cassidy A, Field JK. Incorporation of a genetic factor into an epidemiologic model for prediction of individual risk of lung cancer: the Liverpool Lung Project. Cancer Prev Res (Phila). 2010; 3:664–9. https://doi.org/10.1158/1940-6207.CAPR-09-0141. [PubMed]
31. Hoggart C, Brennan P, Tjonneland A, Vogel U, Overvad K, Ostergaard JN, Kaaks R, Canzian F, Boeing H, Steffen A, Trichopoulou A, Bamia C, Trichopoulos D, et al. A risk model for lung cancer incidence. Cancer Prev Res (Phila). 2012; 5:834–46. https://doi.org/10.1158/1940-6207.CAPR-11-0237. [PubMed]
32. Li H, Yang L, Zhao X, Wang J, Qian J, Chen H, Fan W, Liu H, Jin L, Wang W, Lu D. Prediction of lung cancer risk in a Chinese population using a multifactorial genetic model. BMC Med Genet. 2012; 13:118. https://doi.org/10.1186/1471-2350-13-118. [PubMed]

33. Spitz MR, Amos CI, Land S, Wu X, Dong Q, Wenzlaff AS, Schwartz AG. Role of selected genetic variants in lung cancer risk in African Americans. J Thorac Oncol. 2013; 8:391–7. https://doi.org/10.1097/JTO.0b013e318283da29. [PubMed]
34. Weissfeld JL, Lin Y, Lin HM, Kurland BF, Wilson DO, Fuhrman CR, Pennathur A, Romkes M, Nukui T, Yuan JM, Siegfried JM, Diergaarde B. Lung Cancer Risk Prediction Using Common SNPs Located in GWAS-Identified Susceptibility Regions. J Thorac Oncol. 2015; 10:1538–45. https://doi.org/10.1097/JTO.0000000000000666. [PubMed]
35. Zhu M, Cheng Y, Dai J, Xie L, Jin G, Ma H, Hu Z, Shi Y, Lin D, Shen H. [Genome-wide association study based risk prediction model in predicting lung cancer risk in Chinese]. Zhonghua Liu Xing Bing Xue Za Zhi. 2015; 36:1047–52. [PubMed]
36. Marcus MW, Raji OY, Duffy SW, Young RP, Hopkins RJ, Field JK. Incorporating epistasis interaction of genetic susceptibility single nucleotide polymorphisms in a lung cancer risk prediction model. Int J Oncol. 2016; 49:361–70. https://doi.org/10.3892/ijo.2016.3499. [PubMed]
37. Qian DC, Han Y, Byun J, Shin HR, Hung RJ, McLaughlin JR, Landi MT, Seminara D, Amos CI. A Novel Pathway-Based Approach Improves Lung Cancer Risk Prediction Using Germline Genetic Variations. Cancer Epidemiol Biomarkers Prev. 2016; 25:1208–15. https://doi.org/10.1158/1055-9965.EPI-15-1318. [PubMed]
